# A Swift and Dynamic Strategy to Expand Emergency Department Capacity for COVID-19

**DOI:** 10.1017/dmp.2020.430

**Published:** 2020-11-04

**Authors:** Dennis G. Barten, Renske W.J. Kusters, Nathalie A.L.R. Peters

**Affiliations:** Department of Emergency Medicine, VieCuri Medical Center, Venlo, The Netherlands

**Keywords:** acute medical unit, COVID-19, disaster preparedness, emergency department, pandemic

## Abstract

Emergency departments (EDs) worldwide struggled to prepare for coronavirus disease 2019 (COVID-19) patient surge and to simultaneously preserve sufficient capacity for “regular” emergency care. While many hospitals used costly shelter facilities, it was decided to merge the acute medical unit (AMU) and the ED. The conjoined AMU-ED was segregated into a high-risk and a low-risk area to maintain continuity of emergency care. This strategy allowed for a feasible, swift, and dynamic expansion of ED capacity without the need for external tent facilities. This report details on the technical execution and discusses the pearls and potential pitfalls of this expansion strategy. Although ED preparedness for pandemics may be determined by local factors, such as hospital size, ED census, and primary health-care efficacy, the conjoined AMU-ED strategy may be a potential model for other EDs.

The coronavirus disease 2019 (COVID-19) pandemic, caused by severe acute respiratory syndrome coronavirus 2 (SARS-CoV-2), is and has been an incredible stress test for emergency departments (EDs) worldwide. ED preparation for COVID-19 patient surge while preserving sufficient capacity for the “usual” emergencies is a challenging task. In many countries, emergency medical systems are struggling with increasing patient volumes that exceed clinical and ED capacity.^1^ Reports on how EDs anticipated to this public health emergency are limited.^2-4^

In the Netherlands, the first case of COVID-19 was identified on February 27, 2020. As of August 27, there have been 68,114 confirmed cases of infection (of which 12,126 were hospitalized) and 6215 confirmed COVID-19-deaths.^5^ The adherence area of our hospital is among the hardest hit regions in the Netherlands, with COVID-19 admission rates ranging from 90 to 330 per 100,000 inhabitants.^5^

In late February, our hospital began preparing for a possible COVID-19 surge, which included a strategy to control an increased demand for emergency care. It was decided to merge the ED with the acute medical unit (AMU) to allow a segregation of our ED into a high-risk and a low-risk area, without compromising continuity of emergency care. This strategy made the need for external medical shelters redundant, and it is expected to be dynamically deployed in possible future waves of this pandemic. Our response to COVID-19 may, therefore, be helpful for other EDs preparing for this or future pandemics.

## Setting

VieCuri Medical Center is a 500-bed teaching hospital with a level 2 trauma center and center for percutaneous interventions. The hospital serves a population of 280,000 in a rural area in the south-eastern part of the Netherlands and has an annual ED census of 25,000 patients. The ED contains 11 separate units, including 1 resuscitation bay, 2 trauma bays, and a 2 bed fast-track unit. The hospital is provided with an AMU, which is located directly adjacent to the ED. It consists of 5 separate rooms, accommodating 12 patient beds. The AMU is staffed and equipped to provide multidisciplinary and medical specialist assessment, care, and treatment for all kinds of acute medical conditions.^6^ If admitted, patients are either discharged within 24 h or transferred to specific specialty wards. AMUs are widely implemented in Australia, the United Kingdom, the Netherlands, and Scandinavia.

## Intervention

In late February, shortly after the first case of COVID-19 was identified in the Netherlands, a hospital outbreak management team was formed. In anticipation of increasing patient volumes and possibly prolonged ED throughput times (caused by higher patient acuity and/or limited hospital bed capacity), it was decided to merge the AMU with the ED to expand ED capacity. This merged AMU-ED was initiated on March 17, 2020. For mass casualty incidents, our hospital disaster plan (HDP) already designated the AMU for the care of the secondary or delayed triage category. However, in case of an infectious disease outbreak, this designation was less evident. Although not deployed as an expanded ED, it was not the first time that the AMU served the hospital in times of crisis. During the aftermath of a collapsed ED ceiling in 2017, the AMU was rapidly set up as a temporary ED.^7^

**Figure 1.**
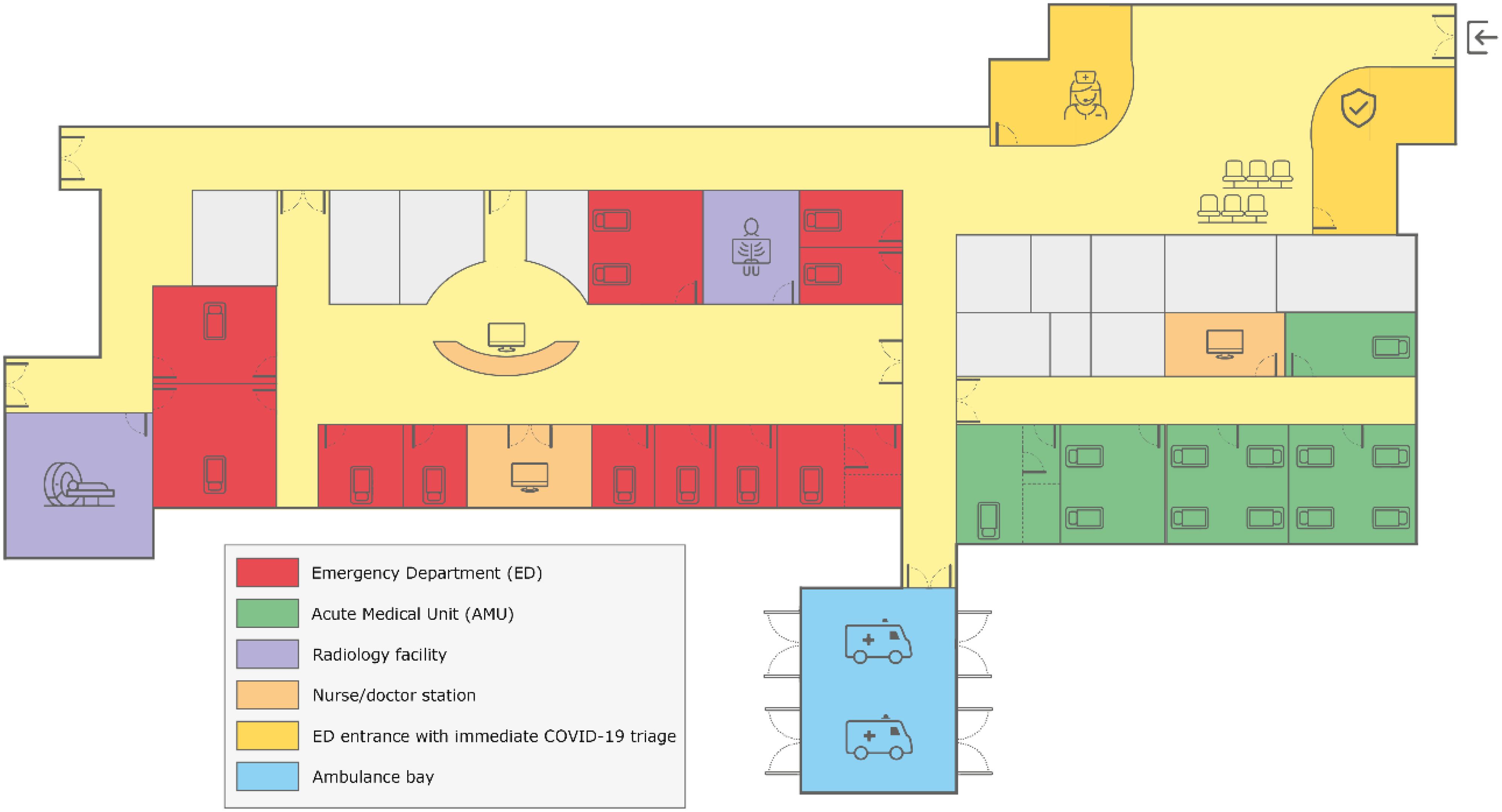
Descriptive floor plan of our AMU (Green area) directly adjacent to our ED (Red area), during the pandemic phase designated into high-risk (Red) and low-risk (Green area). AMU and ED are directly accessible for walk-in patients and ambulance patients. Radiology facilities are located in the ED.

**Figure 2.**
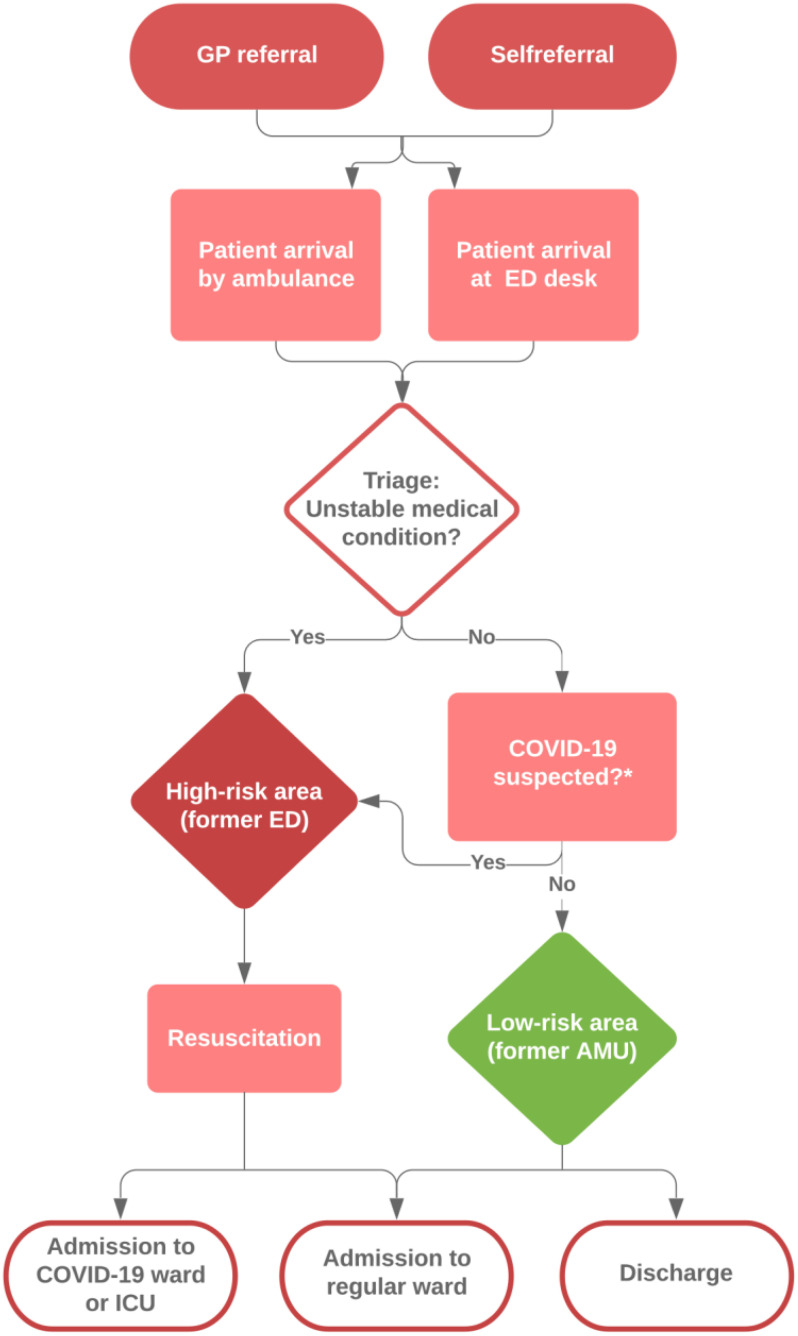
Patient flow chart during the pandemic phase of the COVID-19 crisis. GP, general practitioner; ICU, intensive care unit. *Based on the latest guidelines, concerning symptoms, known COVID-19 contacts, and travel history.

Conjoining the ED and AMU (Figure 1) resulted in a doubled ED capacity in terms of space, and it enabled the segregation of a low- and high-risk area. Pretriage screening was applied for possible COVID-19 infection based on latest guidelines concerning symptoms, known COVID-19 contacts, and travel history before entering the AMU-ED (Figure 2). The former ED was transformed into a high-risk area, where the separate units allowed the assessment of suspected COVID-19 cases in strict isolation. Patients with unstable medical conditions were also assigned to the high-risk area. The former AMU (largely equipped with shared patient rooms) was used for low-risk patients who did not require strict isolation. AMU nursing staff was employed at the low-risk area, where they were supervised by ED nurses. The high-risk area was fully staffed by ED nurses. Both the low- and high-risk areas had 1 coordinating nurse, and the conjoined AMU-ED was led by 1 emergency physician. Personal protective equipment was used in both low- and high-risk areas according to the latest guidelines. Changes in ED and clinical capacity are shown in Table 1.

**Table 1.** Hospital bed capacity: baseline situation versus COVID-19 crisis

	Baseline	COVID-19 Crisis^a^
ED	12	24^c^
AMU	12	0
Intensive care unit^b^	12	32
Proven COVID-19 cohort	0	96
Suspected COVID-19 cohort	0	24

a Maximum capacity.

b Only ICU beds with mechanical ventilators included; during COVID-19 crisis extra ventilator-bed were created in the recovery department and in an unoccupied ICU.

c Represents conjoined AMU-ED.

The AMU-ED strategy was closely monitored from the start, focusing on clinical capacity, throughput, staff, and personal protective equipment. Alternative scenarios, such as the possible use of an external tent facility, were continuously explored and planned for. However, creating sufficient ED capacity within a safe hospital environment had been an important success criterion during the internal hospital disaster in the past, being 1 of the main reasons to merge the AMU with the ED instead of using an external tent facility. A *strengths, weaknesses, opportunities, and threats* (SWOT) analysis of the AMU-ED strategy is shown in Table 2. Nonetheless, preparations were made in an early stage to set up an additional external tent facility within 48 h in case clinical capacity of the expanded ED would be overwhelmed. During the initial COVID-19 surge from March 17 to April 17, 2020, a total of 634 patients were triaged to the AMU (low-risk) and 780 patients to the ED (high-risk or unstable). Of the patients triaged to the AMU, 9 (1.4%) later received a COVID-19 diagnosis, which could be linked to their index ED visit. All of these patients presented with nonspecific complaints and did not meet the case definition during the AMU-ED visit.

**Table 2.** Strengths, weaknesses, opportunities, and threats (SWOT) analysis of the AMU-ED strategy

Strengths	Weaknesses	Opportunities	Threats
Ability to provide emergency care within the same area and within the hospital’s walls.	AMU staff does not usually care for ED patients. Therefore, they have to be supervised/assisted by ED staff.	Avoiding the need for an external shelter facility may reduce costs and possible waste of resources.	Maximum capacity of the conjoined AMU-ED may not be sufficient during a pandemic.
Staff is familiar with AMU and ED area and its processes.	ED staff has to be distributed over 2 departments, but not in the extend required for an external facility.	Applying the AMU-ED strategy does not exclude the set up of an external shelter facility if indicated.	Unsure whether segregation of cases with pretriage screening is actually safe.
The AMU has successfully served as a temporary ED in the past.	Unstable patients cannot be treated in the AMU area.	The set up of an external shelter facility may lead to social anxiety (“the hospital is not in control anymore”), whilst the AMU-ED strategy is not visible on the outside.	Triage criteria continue to evolve.
Climate control issues are not expected.			Clinical AMU capacity (both staff and space) cannot be used for admissions.
AMU-ED can be dynamically deployed and used for future waves of this pandemic.			

When the COVID-19 surge declined, the original function of the AMU was gradually restored. Initially, all acute patients, both high- and low-risk, were assessed in isolation in the ED. In case of undercapacity of the ED, patients were also assessed in the AMU. Eventually, the AMU was gradually reopened for acute admissions, while maintaining capacity for ED assessment.

During the entire crisis, the AMU-ED capacity was not exceeded. This made the need for external medical shelters redundant. The conjoined AMU-ED strategy has proven to be successful and can be rapidly implemented. Therefore, in case of possible future waves of this pandemic, the AMU-ED approach may be executed again, either partially or completely.

## Discussion

While many hospitals used costly shelter facilities, our relatively simple strategy allowed to maintain ED capacity within the hospital’s walls. Although ED preparedness for pandemics may be determined by local factors, such as hospital size, ED census, and primary health-care efficacy, we believe that the conjoined AMU-ED strategy may be successfully applied in other hospitals too, as long as they are equipped with an AMU or a similar intermediate care unit close to the ED. AMUs are typically situated in the vicinity of the ED, and their size is usually aligned with ED census.

Although there are successful examples of outside structures used to expand ED capacity,^2,3^ there are also concerns. First, it is challenging to align and coordinate emergency care that is delivered on separate locations, mainly because adequately trained ED staff has to be distributed, which makes team management more difficult. Second, it requires extra staffing creativity and flexibility of those already under pressure. Third, it may be challenging to cope with climate control, particularly in case of extreme weather events. Fourth, outside shelters may be considered substandard care or may lead to social anxiety. Finally, its use is associated with high costs and possible waste of resources, because preserving the shelters for future pandemic surges is not always feasible.

There are some limitations of this report and strategy. Most importantly, this is a retrospective single site experience. Furthermore, detection of possible COVID-19 cases was challenging as triage criteria continued to evolve. In the early phase of the pandemic, the Dutch case definition was relatively strict, missing COVID-19 patients that presented with nonspecific complaints. Therefore, most of the 9 undertriaged patients presented in the early days of the conjoined AMU-ED. This miss rate of 1.4% is similar to the findings of a Singaporese study,^8^ and it underlines the importance of continued vigilance at the frontline to reduce the likelihood of nocosomial transmission. Maximum AMU-ED capacity was never exceeded. However, in this perspective, it should be noted that, during the early phase of the COVID-19 pandemic, ED use was lower than usual.^9^ This phenomenon has been observed worldwide and in several health-care settings. Normal ED use in addition to the COVID-19 surge could possibly have led to overwhelmed capacity.

## Conclusions

Conjoining the ED with an adjacent AMU or similar intermediate care unit allows for a feasible, rapid, and dynamic expansion of ED capacity during a pandemic and reduces the need for external tent facilities.
